# Recovery from Salinity and Drought Stress in the Perennial *Sarcocornia fruticosa* vs. the Annual *Salicornia europaea* and *S. veneta*

**DOI:** 10.3390/plants11081058

**Published:** 2022-04-13

**Authors:** Roberta Calone, Diana-Maria Mircea, Sara González-Orenga, Monica Boscaiu, Carla Lambertini, Lorenzo Barbanti, Oscar Vicente

**Affiliations:** 1 Department of Agricultural and Food Sciences, Alma Mater Studiorum, University of Bologna, Viale Fanin 44, 40127 Bologna, Italy; roberta.calone3@unibo.it; 2 Institute for Conservation and Improvement of Valencian Agrodiversity (COMAV), Universitat Politècnica de València, Camino de Vera 14, 46022 Valencia, Spain; dmircea@doctor.upv.es (D.-M.M.); sagonor@doctor.upv.es (S.G.-O.); ovicente@upvnet.upv.es (O.V.); 3 Department of Horticulture and Landscape, University of Agricultural Sciences and Veterinary Medicine of Cluj-Napoca, 3-5 Manastur St., 400372 Cluj-Napoca, Romania; 4 Mediterranean Agroforestry Institute (IAM), Universitat Politècnica de València, Camino de Vera 14, 46022 Valencia, Spain; mobosnea@eaf.upv.es; 5 Dipartimento di Bioscienze, Università di Milano, Via Celoria 26, 20133 Milano, Italy; carla.lambertini@unimi.it

**Keywords:** *Sarcocornia fruticosa*, *Salicornia europaea*, *Salicornia veneta*, halophytes, salt stress, drought stress, stress recovery, osmolytes, ion transport, oxidative stress markers

## Abstract

Current agricultural problems, such as the decline of freshwater and fertile land, foster saline agriculture development. *Salicornia* and *Sarcocornia* species, with a long history of human consumption, are ideal models for developing halophyte crops. A greenhouse experiment was set up to compare the response of the perennial *Sarcocornia fruticosa* and the two annual *Salicornia europaea* and *S. veneta* to 30 days of salt stress (watering with 700 mM NaCl) and water deficit (complete withholding of irrigation) separate treatments, followed by 15 days of recovery. The three species showed high tolerance to salt stress, based on the accumulation of ions (Na^+^, Cl^−^, Ca^2+^) in the shoots and the synthesis of organic osmolytes. These defence mechanisms were partly constitutive, as active ion transport to the shoots and high levels of glycine betaine were also observed in non-stressed plants. The three halophytes were sensitive to water stress, albeit *S. fruticosa* to a lesser extent. In fact, *S. fruticosa* showed a lower reduction in shoot fresh weight than *S. europaea* or *S. veneta*, no degradation of photosynthetic pigments, a significant increase in glycine betaine contents, and full recovery after the water stress treatment. The observed differences could be due to a better adaptation of *S. fruticosa* to a drier natural habitat, as compared to the two *Salicornia* species. However, a more gradual stress-induced senescence in the perennial *S. fruticosa* may contribute to greater drought tolerance in this species.

## 1. Introduction

In response to the current increase in world population, agriculture is called to address two major but opposite needs: increasing food production while decreasing its negative environmental impacts. Boosting food security through sustainable agricultural practices represents a priority objective for the 2030 Agenda for Sustainable Development [1], a goal that, to date, is even more urgent, considering that, in 2020, the number of undernourished people worldwide has increased by 83–132 million due to the COVID-19 pandemic [2]. However, the growing competition for land and water caused by the dramatic expansion of cities [3], in conjunction with the increasingly recurrent phenomena of soil erosion, water scarcity, and loss of agrobiodiversity, are posing serious obstacles to achieving this objective.

The Mediterranean basin is amongst the areas most threatened by salinisation in the world due to climate change [4]. According to the Intergovernmental Panel on Climate Change, in the Mediterranean region, temperatures will rise by 2–4 °C, and rainfall will decrease between 4% and 30% by 2050 [5], whereas sea level is expected to increase by approximately 35 cm by 2100 [6]. The projected climate changes will also exacerbate the salt accumulation processes driven by seawater intrusion in the coastal shallow aquifers, which in turn will constrain soil fertility and crop productivity.

In 2009, the World Bank introduced the concept of climate-smart agriculture (CSA), referring to an integrated approach to address the complex nexus of climate change, food security, and sustainable development [7]. Today, the FAO Strategic Framework 2022–2031 considers the transition to CSA imperative to improve agricultural resilience and productivity and lower its climate footprint and costs [8]. The CSA approach is implemented through three priority lines of action: firstly, boosting sustainable agricultural production to support increased incomes and food security; secondly, increasing agroecosystems’ adaptive capacity; and thirdly, reducing greenhouse gas emissions while increasing carbon sequestration [9].

The CSA applications are context-specific, depending on the local socio-political, financial, and environmental context, and encourage the integration of new technologies and practices such as precision farming tools, decision support systems for land and water management, conservative and organic crop practices, integrated pest and disease management, and the introduction of drought-, salt-, and flood-tolerant crops [10]. In this last regard, the Mediterranean region represents a precious hotspot of biodiversity, with a remarkable richness in cultivated and native wild plants that have adapted to various unfavourable conditions such as prolonged drought, salinity, and flooding.

Halophytes are extremophile plants that can tolerate harsh conditions and salinity levels toxic to most plants. Within the CSA framework, the study of halophytes’ stress tolerance mechanisms is an outlooking strategy for improving crop resilience to environmental stress. Besides providing valuable scientific models, these plants can be cultivated for the direct production of food, fodder, biomass and medicinal compounds, as well as for soil phytoremediation, carbon sequestration, and landscaping purposes, including the recovery of marginal saline soils and water [11,12]. About 1100 halophyte species occur in the Mediterranean Basin, when considered in its broadest meaning, i.e., from the Aral Sea to the Atlantic Ocean [13]. Taxonomical, biological, and ecological diversity is high here, and there are traditional and new potential uses of these plants.

The subfamily *Salicornioideae* includes around 100 species of succulent halophytes, the *Sarcocornia*/*Salicornia* lineage being one of the most important in terms of species diversity [14]. This lineage consists of hygro-halophytes diversified during the Middle Miocene [15] and was confirmed by transcribed spacer (ITS) and atpB–rbcL spacer sequences as monophyletic, being clearly separated from other taxa [15]. Molecular phylogenetic studies based on external transcribed spacer (ETS) sequence revealed that this lineage comprises three primary clades: *Salicornia*, American-Eurasian *Sarcocornia*, and South African-Australian *Sarcocornia* [16]. The genus *Sarcocornia* A.J. Scott was separated from *Salicornia* L. and *Arthrocnemum* Moq. on the basis of morphological characters [17]. The *Salicornia* and *Sarcocornia* genera are morphologically similar and can be distinguished only by inflorescence characters and their life form, the former including only annuals and the latter only perennials. *Salicornia* is clearly a monophyletic genus, as revealed by ETS sequence data [16], whereas *Sarcocornia* remains unresolved as possibly paraphyletic [14]. Annual *Salicornia* species evolved from the perennial *Sarcocornia* during Miocene, and their high self-fertility allowed their rapid expansion, colonising coastal and inland remote habitats [14,16].

Three species of the *Sarcocornia*/*Salicornia* lineage were selected for this study. *Salicornia europaea* L. belongs to a diploid clade including genotypes that show a wide geographical distribution. *S. veneta* Pign. et Lausi is a member of the well-supported monophyletic group of *Salicornia dolichostachya* Moss with very little genetic variation among its taxa [16,18]. The species is endemic to NE Italy in the area of the Lagoon of Venice and West Slovenia and is classified as vulnerable according to the UICN criteria [19]. The third species under study, *Sarcocornia fruticosa* (L.) A.J. Scott with Mediterranean distribution, belongs to the Eurasian clade of *Sarcocornia* [14]. The three species are morphologically similar, with succulent and articulate stems, reduced leaves, and inflorescences of minute reduced flowers. Their young, fleshy tips are edible and commercialised with the name of “samphire”, “sea asparagus”, “pickleweed”, or “poor man’s asparagus” [20]. Thanks to the crunchy texture and salty taste, their succulent shoots are highly appreciated in gourmet cuisine [21,22,23]. Moreover, they are a good source of fibre, antioxidants, and anti-inflammatory metabolites, such as vitamin C and polyphenolic compounds, making them an ideal nutraceutical supplement [23,24]. These species are also appreciated as oil-seed crops. Indeed, oil extracted from their seeds is rich in polyunsaturated fatty acids, particularly oleic and linoleic acid, having valuable health properties [25]. Furthermore, these species can produce high amounts of biomass rich in lignocellulosic materials suitable for bioethanol production [26]. The high biomass production, combined with the high phytoextraction capacity, also makes these species very attractive for the phytoremediation of saline and heavy metal-contaminated soils [27]. Finally, several studies have demonstrated their suitability for the regreening of marginal areas to increase carbon sequestration and relieve soil erosion [28,29].

Without salt glands or salt bladders, the strategy of glassworts to tolerate the ionic and osmotic components of salt stress relies largely on the massive accumulation and vacuolar compartmentalisation of Na^+^ and Cl^−^ [30,31,32,33], which allow them to maintain the osmotic potential necessary to drive water uptake into cells while preventing ion-related cytotoxic effects. Moreover, they have evolved the ability to increase succulence in shoots diluting the accumulated ions [34], synthesise compatible solutes for osmotic adjustment, especially glycine betaine [34,35,36,37], produce ROS-scavenging enzymes and compounds [38,39], maintain high K-Na selectivity [33], and effectively regulate ammonium detoxification processes under stress conditions [40]. Furthermore, glassworts have the ability to transit from green to reddish colouration through the accumulation of red-violet pigments and betacyanins, which allow them to cope with excessive light energy in the photosystems when the plants experience osmotic stress and photosynthesis declines by dissipating excess excitation energy into heat [41].

In their natural habitats, halophytes are subjected to wide seasonal oscillations in precipitations and temperature, and therefore in soil moisture and salinity, which result in periods of high and low stress intensity that alternate during the year [42]. Significantly stressful conditions at the field level, however, are often only transient and rarely cause plant death as more favourable conditions usually return, although they often result in reduced crop yield [43]. However, basic studies on stress tolerance in halophytes have generally focused on their responses to different applied stress treatments, and very little is known on the equally important mechanisms of stress recovery, which are essential for ensuring sustainable crop production under intermittent stress events.

The focus of the present study was to analyse differences between the three aforementioned Salicornioideae species in their responses to stress and stress recovery treatments, which could be due to differences in the plants’ life cycle or native environments. For this, we determined growth parameters in plants of the investigated species after applying controlled salt and water deficit treatments in a greenhouse, followed by irrigation with non-saline water. To obtain insights into their stress tolerance mechanisms, growth responses were correlated with changes in the levels of specific biochemical stress markers, such as photosynthetic pigments, different mono and divalent ions and organic osmolytes, oxidative stress markers, and antioxidant compounds.

## 2. Results

### 2.1. Substrate Electric Conductivity and Moisture

**Figure 1:**
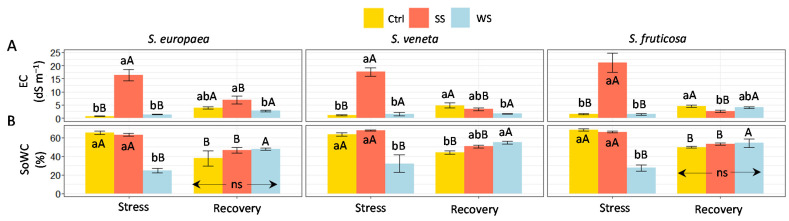
Effect of 30 days of stress treatments (Stress), followed by watering with non-saline water for 15 days (Recovery) on (**A**) Substrate electrical conductivity (soil EC) and (**B**) water content (soil WC). Ctrl, control; SS, salt stress (watering with 700 mM NaCl); WS, water stress (complete withholding of irrigation). For each species and sampling (Stress or Recovery), different lowercase letters over the bars indicate significant differences between treatments (Ctrl, SS, and WS) at *p* ≤ 0.05. Different uppercase letters indicate significant differences between the two sampling times (Stress and Recovery) for each species and treatment, at *p* ≤ 0.05. Vertical bars indicate standard error (*n* = 4). ns: non-significant.

During the stress period, the substrate electric conductivity (EC) increased significantly in the pots subjected to salt stress, reaching over 15 dS m^−1^ for all three halophytes, with a maximum of 21 dS m^−1^ in *S. fruticosa*, whereas the water stress treatment did not cause any change in the control EC values (Figure 1A). After 15 days of watering the pots with non-saline water (‘recovery’ treatment), the substrate EC in salt-treated pots decreased to control values (for *S. europaea* and *S. veneta*) or even slightly (but significantly) below the control for *S. fruticosa*. However, substrate salinity in the pots previously subjected to the withholding of irrigation remained similar to the controls after recovery (Figure 1A).

Contrary to the EC data, the substrate water content, with control values of about 65% for all three halophytes, was not affected by the salt treatment; however, soil moisture decreased significantly under water deficit conditions, down to between 25 and 30%, depending on the species (Figure 1B). After recovery from water stress, substrate moisture increased to reach values equal or even higher (in *S. veneta*) than the controls, whereas recovery from salt stress did not alter the soil water content when compared to the corresponding controls (Figure 1B).

### 2.2. Plant Growth

Plant height and the number of branches were measured in all plants at the beginning (T0) and every 15 days during the experiments; that is, after 15 and 30 days of water or salt stress and at the end of the ‘recovery’ treatment (Table 1). Both parameters increased significantly during the stress treatments in control and stressed plants. The salt treatment did not cause significant growth inhibition in any of the three species. In contrast, compared to the control, water deficit induced a significant plant height reduction in the two *Salicornia* species and also a reduction (down to 57% of the control) in the number of branches in *S. europaea*. However, this inhibitory effect was only observed after 30 days of withholding irrigation, not at day 15 of the treatment (Table 1). These data indicate a strong tolerance of the three species to salinity, even at very high salt concentrations (700 mM NaCl), and a slightly higher drought sensitivity of the two *Salicornia* species compared to *Sarcocornia fruticosa*.

After 15 days of recovery, the plant height and the number of branches of *S. europaea* and *S. veneta* plants were statistically homogeneous in all treatments (control and water and salt stress); the same result was observed for plant height in *S. fruticosa*. The number of branches increased during recovery in the latter species but to a lesser extent in the previously stressed plants, which did not reach the control values (Table 1).

**Figure 2:**
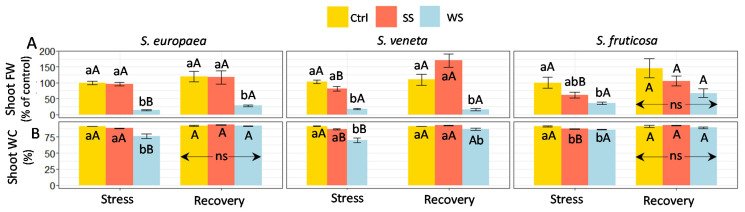
Effect of 30 days of stress treatments (Stress), followed by watering with non-saline water for 15 days (Recovery) on (**A**) shoot fresh weight (FW) and (**B**) shoot water content (SWC) in the three halophytes. Ctrl, control; SS, salt stress (watering with 700 mM NaCl); WS, water stress (complete withholding of irrigation). For each species and sampling (Stress or Recovery), different lowercase letters over the bars indicate significant differences between treatments (Ctrl, SS, and WS), whereas different uppercase letters indicate significant differences between the two samplings (Stress and Recovery) for each species and treatment, at *p* ≤ 0.05. Vertical bars indicate standard error (*n* = 4). Values in (**A**) are shown as percentages of shoot FW of control plants (Ctrl, Stress), taken as 100%; the corresponding absolute values for *S. europaea*, *S. veneta*, and *S.fruticosa* were 13.3, 10.1, and 5.6 g plant^−1^, respectively. ns: non-significant.

After the stress and recovery periods, plants were harvested to determine shoot fresh weight (FW) and water content percentage (WC) as the most reliable parameters to assess the treatment effects on plant growth. Salt stress did not affect the shoot FW or WC of the *Salicornia* species significantly, whereas *S. fruticosa* plants appeared to be slightly more affected, with a more accentuated (but still non-significant) reduction in the mean FW and a slight (but significant) reduction in WC (Figure 2A,B). On the other hand, water stress strongly reduced shoot FW in the three species (Figure 2A), partly due to plant dehydration, as it was accompanied by a small but significant WC decrease compared to the control plants (Figure 2B).

After recovery, the salt-stressed plants of the three halophytes maintained a shoot FW and WC similar to their corresponding controls. However, watering with non-saline water had distinct effects on plants previously subjected to water deficit, depending on the species. Thus, *S. europaea* plants showed a significant increase in FW upon recovery, but with values still well below those of the control plants and the complete rehydration of the shoots; in contrast, no significant effects were observed in *S. veneta*. Only in *S. fruticosa* did shoot FW not show any statistically significant differences from the control after recovery, although the mean value was lower (Figure 2). Therefore, confirming the measurements of other growth parameters, *S. fruticosa* appears to be more tolerant to drought than the *Salicornia* species and also shows better recovery from the water deficit treatment.

### 2.3. Photosynthetic Pigments

**Figure 3:**
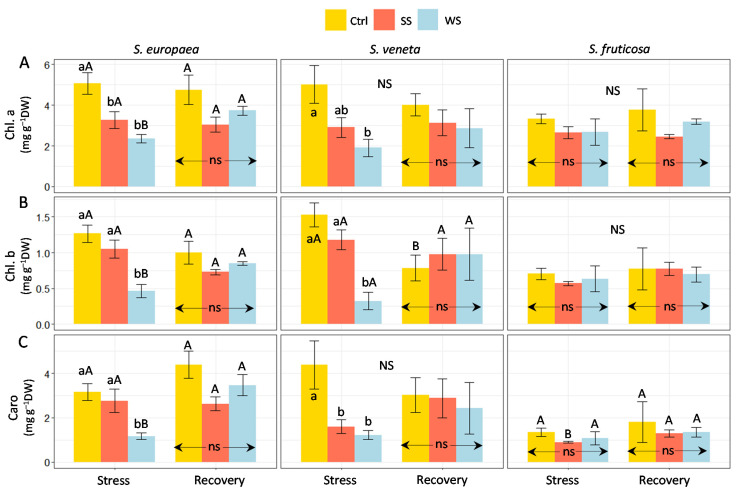
Effect of 30 days of stress treatments (Stress), followed by watering with non-saline water for 15 days (Recovery) on (**A**) chlorophyll a (Chl. a), (**B**) chlorophyll b (Chl. b), and (**C**) carotenoids (Caro) in the three halophytes. Ctrl, control; SS, salt stress (watering with 700 mM NaCl); WS, water stress (complete withholding of irrigation). For each species and sampling (Stress or Recovery), different lowercase letters over the bars indicate significant differences between treatments (Ctrl, SS, and WS), at *p* ≤ 0.05; ns: non-significant. Different uppercase letters indicate significant differences between the two samplings (Stress and Recovery) for each species and treatment, at *p* ≤ 0.05; NS: non-significant. Vertical bars indicate standard error (*n* = 4).

Mean values of photosynthetic pigment contents showed a decreasing trend in response to the salt treatment in plants of the two annual *Salicornia* species (Figure 3); however, the differences with the non-stressed plants were only significant for chlorophyll a (Chl. a) in *S. europaea* (Figure 3A) and carotenoids (Caro) in *S. veneta* (Figure 3C), whereas no variations in chlorophyll b (Chl. b), the second most abundant chlorophyll in oxygenic photosynthetic organisms, were recorded. After irrigation with non-saline water, no significant differences with the controls were found for any pigment. In contrast, water deficit caused a significant reduction in the levels of the three pigments in both annual species; in all cases, mean pigment contents increased after the recovery treatment, reaching values not significantly different from the controls. On the other hand, in the perennial *S. fruticosa,* neither salt nor water stress induced any significant variation in pigment concentrations, and the recovery treatment had no effect, except for a slight yet significant increase in Caro levels in salt-treated plants. However, it should be mentioned that the pigment levels in the *S. fruticosa* control plants were lower than those determined in *S. europaea* and *S. veneta* (Figure 3). These responses agree with the observed stress-induced changes in growth parameters, confirming the high salt tolerance of the three species, the relatively higher drought tolerance of *S. fruticosa* compared to the annual species, and the effectiveness of the recovery treatment.

### 2.4. Ion Accumulation

**Figure 4:**
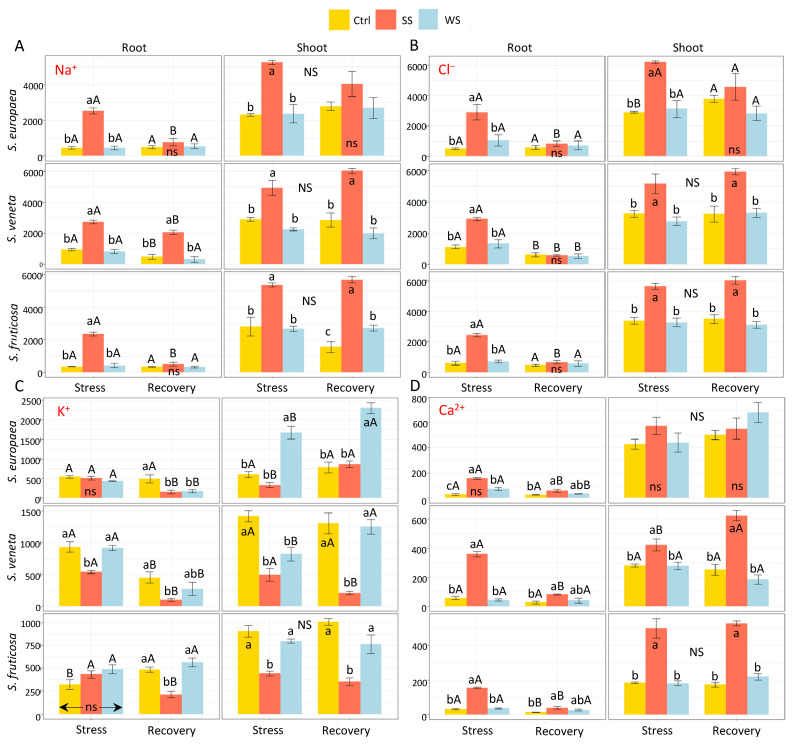
Effect of 30 days of stress treatments (Stress) followed by watering with non-saline water for 15 days (Recovery) on the root and shoot concentration (in μmol g^−1^ DW) of ions: (**A**) sodium (Na^+^), (**B**) chloride (Cl^−^), (**C**) potassium (K^+^), and (**D**) calcium (Ca^2+^) in the three halophytes. Ctrl, control; SS, salt stress (watering with 700 mM NaCl); WS, water stress (complete withholding of irrigation). For each species and sampling (Stress or Recovery), different lowercase letters over the bars indicate significant differences between treatments (Ctrl, SS, and WS), at *p* ≤ 0.05; ns: non-significant. Different uppercase letters indicate significant differences between the two samplings (Stress and Recovery) for each species and treatment, at *p* ≤ 0.05; NS: non-significant. Vertical bars indicate standard error (*n* = 4).

Root and shoot Na^+^ and Cl^−^ concentrations increased significantly in response to the salt stress treatment in the three halophytes, as expected, whereas water deficit did not have any effect on the ions levels. The recovery treatment reduced the contents of both ions in roots of salt-stressed plants down to control levels, except for Na^+^ in *S. veneta*, which showed a still significant but less accentuated decrease. In contrast, no differences were observed in shoot Na^+^ or Cl^−^ contents before and after recovery, except *for S. europaea*, in which Cl^−^ content increased slightly but significantly in the control. Under all tested conditions, the concentrations of both ions were substantially higher in shoots than in roots (Figure 4A,B).

Variations of K^+^ concentrations showed different patterns, depending on the species and the treatments (Figure 4C). First, control levels in the roots of non-stressed plants differed substantially between species, being the highest in *S. veneta*—about 1.7-fold higher than in *S. europaea* and three-fold higher than in *S. fruticosa*, approximately. Shoot K^+^ contents were similar to those in roots in *S. europaea*, whereas they were higher in shoots than in roots in *S. veneta* and *S. fruticosa*. The stress treatments did not cause changes in the root K^+^ concentration, except for the significant decrease observed in salt-stressed *S. veneta* plants. At the shoot level, mean K^+^ concentrations decreased upon salt treatment, although the difference with the control was non-significant in *S. europaea*. Under water stress, K^+^ contents increased, decreased, and remained the same as in the controls in *S. europaea*, *S. veneta*, and *S. fruticosa*, respectively. After recovery, K^+^ concentrations were generally lower than control values in the roots and shoots of salt-stressed plants and not significantly different from the controls in plants previously subjected to water stress, although some exceptions to this general behaviour were observed in *S. europaea* (Figure 4C).

The patterns of Ca^2+^ variation in the roots of the three species were similar to those observed for Na^+^ and Cl^−^, that is, a significant increase in response to salt stress and no effect of water stress except for an increase in *S. europaea* (Figure 4D). Shoot Ca^2+^ concentration significantly increased in the salt-treated plants of *S. veneta* and *S. fruticosa*, but not of *S. europaea,* with no effect of water stress. After the recovery period, root Ca^2+^ concentration in the salt-stressed plants decreased but remained significanlty higher than in control plants, and was statistically comparable with the water-stressed plants. In shoots, the Ca^2+^ concentration did not vary after recovery, except for an increase in the salt-treated plants of *S. veneta* (Figure 4D).

### 2.5. Osmolytes, Oxidative Stress Markers and Antioxidants

**Figure 5:**
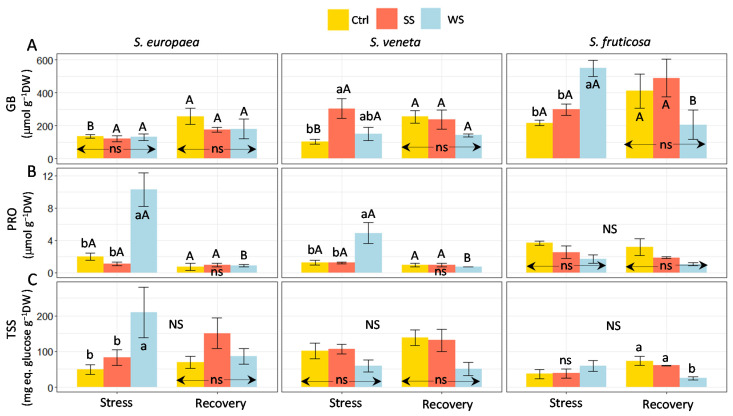
Effect of 30 days of stress treatments (Stress) followed by watering with non-saline water for 15 days (Recovery) on shoot concentration of (**A**) glycine betaine (GB), (**B**) proline (PRO), and (**C**) Total Soluble Sugars (TSS) in the three halophytes. Ctrl, control; SS, salt stress (watering with 700 mM NaCl); WS, water stress (complete withholding of irrigation). For each species and sampling (Stress or Recovery), different lowercase letters over the bars indicate significant differences between treatments (Ctrl, SS, and WS), at *p* ≤ 0.05; ns: non-significant. Different uppercase letters indicate significant differences between the two samplings (Stress and Recovery) for each species and treatment, at *p* ≤ 0.05; NS: non-significant. Vertical bars indicate standard error (*n* = 4).

Common osmolytes, glycine betaine (GB), proline (PRO), and total soluble sugars (TSS) were determined and showed distinct accumulation patterns in the shoots of the selected species (Figure 5). Neither salt stress nor water deficit caused any significant change in GB contents in *S. europaea*; they augmented three-fold over control values in salt-stressed *S. veneta* and about 2.5-fold in *S. fruticosa* plants subjected to water stress. After the recovery period, the GB level increased significantly in non-stressed *S. europaea* and *S. veneta* plants and decreased in those of *S. fruticosa* that underwent the water deficit treatment. Nevertheless, no significant differences between treatments were found in the shoot GB contents of any of the three halophytes after recovery (Figure 5A).

PRO contents did not vary in any species in response to salt stress but increased in the water-stressed plants of *S. europaea* (about five-fold over the control) and, to a lesser extent, *S.veneta* (ca. four-fold). In these two *Salicornia* species, PRO levels decreased to control values after the recovery period, so that, in all cases, the differences between treatments became non-significant. In *S. fruticose*, no variation in PRO contents was observed, for any of the samples, after the stress treatments and after recovery (Figure 5B). Under all experimental conditions, PRO concentrations in molar terms were much lower than those of GB in the three species. GB contents ranged between 100 and more than 500 μmol g^−1^ DW, whereas the maximum measured PRO level (in water-stressed *S. europaea* plants) was only ca. 10 μmol g^−1^ DW (Figure 5A,B).

Only the water-stressed *S. europaea* plants showed a significant increase in shoot TSS levels; all other differences between control and stressed plants in the stress and recovery treatments, or between the two samplings, were non-significant (Figure 5C).

**Figure 6:**
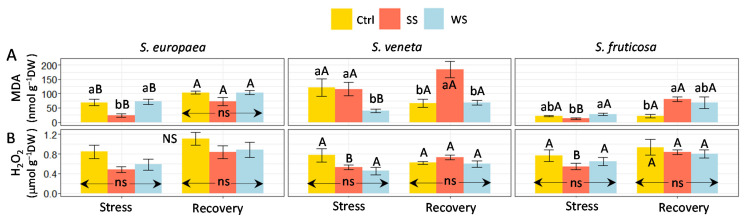
Effect of 30 days of stress treatments (Stress) followed by watering with non-saline water for 15 days (Recovery) on shoot concentration of (**A**) Malondialdehyde (MDA) and (**B**) hydrogen peroxide (H_2_O_2_) in the three halophytes. Ctrl, control; SS, salt stress (watering with 700 mM NaCl); WS, water stress (complete withholding of irrigation). For each species and sampling (Stress or Recovery), different lowercase letters over the bars indicate significant differences between treatments (Ctrl, SS, and WS), at *p* ≤ 0.05; ns: non-significant. Different uppercase letters indicate significant differences between the two samplings (Stress and Recovery) for each species and treatment, at *p* ≤ 0.05; NS: non-significant. Vertical bars indicate standard error (*n* = 4).

To assess the possible generation of secondary oxidative stress in the plants subjected to salt or water stress treatments, the contents of two reliable biochemical markers, malondialdehyde (MDA) and hydrogen peroxide (H_2_O_2_), were determined in the shoots of all plants (Figure 6). No increase in MDA or H_2_O_2_ levels was detected in any of the samples from the stressed plants in relation to the non-stressed controls. MDA contents even decreased in some cases, namely under salt stress in *S. europaea* and under water stress in *S. veneta*. In contrast, no differences in H_2_O_2_ content between stressed and control plants were detected in the three species. A significant increase in MDA concentration was observed after the recovery period in the salt-stressed plants of *S. europaea* and *S. fruticosa* and in the water-stressed plants of *S. europaea* and *S. veneta*. On the other hand, H_2_O_2_ levels increased after recovery in the salt-treated plants of *S. veneta* and *S. fruticosa* (Figure 6).

**Figure 7:**
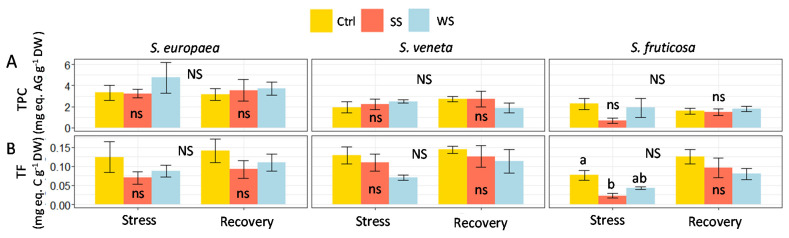
Effect of 30 days of stress treatments (Stress) followed by watering with non-saline water for 15 days (Recovery) on shoot concentration of (**A**) total phenolic compounds (TPC) and (**B**) total flavonoids (TF) in the three halophytes. Ctrl, control; SS, salt stress (watering with 700 mM NaCl); WS, water stress (complete withholding of irrigation). For each species and sampling (Stress or Recovery), different lowercase letters over the bars indicate significant differences between treatments (Ctrl, SS, and WS), at *p* ≤ 0.05; ns: non-significant. Different uppercase letters indicate significant differences between the two samplings (Stress and Recovery) for each species and treatment, at *p* ≤ 0.05. NS: non-significant. Vertical bars indicate standard error (*n* = 4).

In agreement with the lack of a detectable generation of oxidative stress under high salinity and water deficit conditions, the activation of the synthesis of common antioxidant compounds, such as phenolic compounds (TPC) and, particularly, the subgroup of flavonoids (TF), was also not observed. Indeed, differences in TPC and TF contents between treatments during the stress and recovery periods were generally non-significant, except for the TF reduction in response to salt in *S. fruticosa*. Moreover, no differences were detected between samplings for each treatment (Figure 7).

### 2.6. Physiological Traits Relationships and Results of the Multivariate Analysis

**Figure 8:**
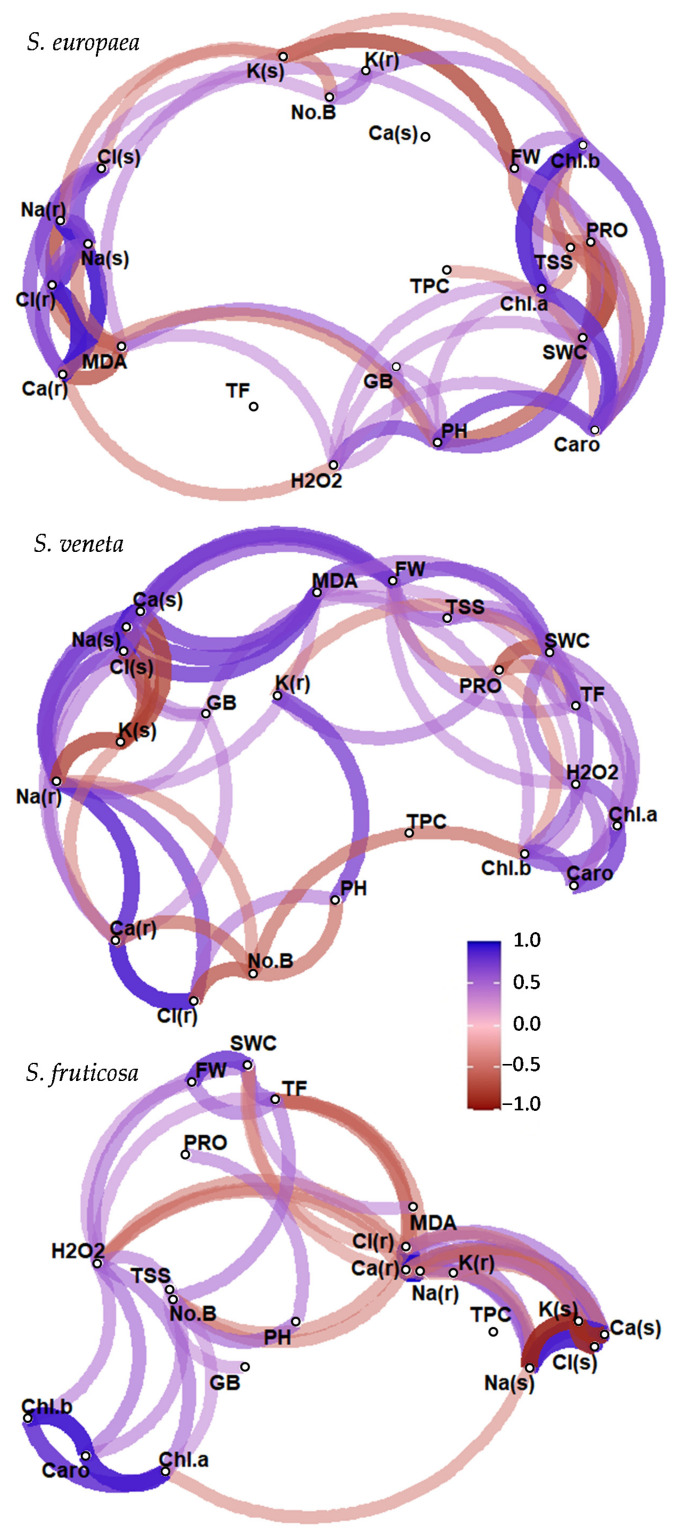
Correlation network diagram showing significant correlations (*p* < 0.05) between the 22 measured traits within each halophyte species, based on the calculation of the Pearson correlation coefficients. Each measured trait represents a node, and highly correlated traits are clustered together. Each path represents a correlation between the two variables it joins. A blue path represents a positive correlation and a red path represents a negative correlation. Only significant correlations are represented. The width and transparency of the line represent the strength of the correlation (wider and less transparent = stronger correlation). Abbreviations: fresh weight (FW), shoot water content (SWC), plant height (PH), number of branches (No.B), chlorophyll a (Chl. a), chlorophyll b (Chl. b), carotenoids (Caro), root sodium concentration (Na(r)), shoot sodium concentration (Na(s)), root chloride concentration (Cl(r)), shoot chloride concentration (Cl(s)), root potassium concentration (K(r)), shoot potassium concentration (K(s)), root calcium concentration (Ca(r)), shoot calcium concentration (Ca(s)), glycine betaine (GB), proline (PRO), total soluble sugars (TSS), malondialdehyde (MDA), hydrogen peroxide (H_2_O_2_), total phenolic compounds (TPC), total flavonoids (TF).

In the three surveyed species, some common trait patterns could be observed (Figure 8). The pigments, namely Chl. a, Chl. B, and Caro, were positively correlated with each other in all three species, indicating their covariation. The potassium shoot concentration, K(s), instead, always resulted in being negatively correlated with Na(r), Na(s), and Cl(s). Plant FW was consistently positively correlated with the shoot water content (SWC), which was positively associated with Chl. a and Caro contents in the two annual plants. Furthermore, SWC in the two *Salicornia* species was negatively correlated with PRO, as the content of this osmolyte mostly increased under water stress, when the plant SWC was the lowest.

The near absence of significant correlations between TPC and other growth-related traits confirmed that salinity and water deficit, under our experimental conditions, did not generate a substantial degree of oxidative stress in the plants.

**Figure 9:**
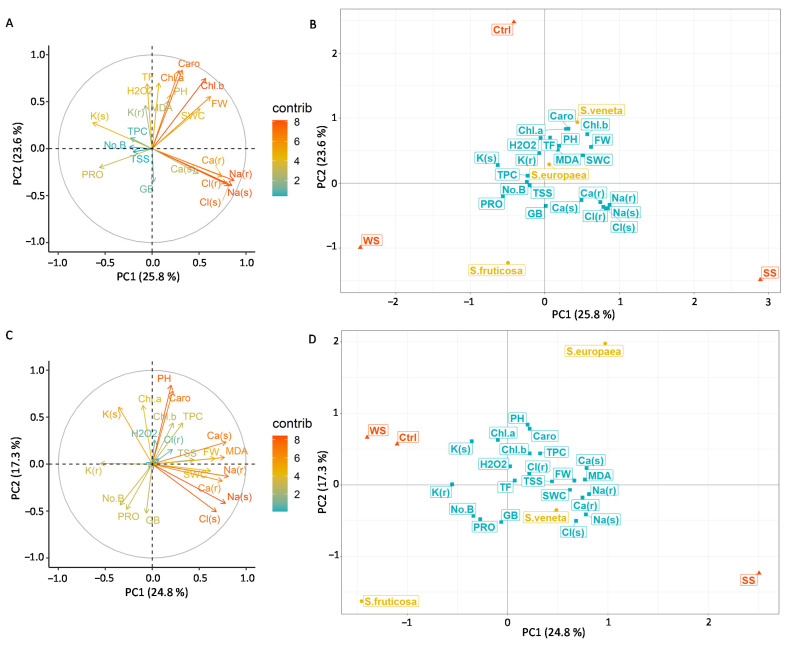
PCA correlation circles of the 22 measured parameters: (**A**) after 30 days of stress treatments (PCAstress) and (**C**) after 15 days of watering with non-saline water (PCArecovery). The increasing arrow lengths and shades of colour from light blue to red indicate the increasing contribution of variables to the definition of the first two principal components. PCA biplot of variables (**B**) after 30 days of stress treatments (Stress) and (**D**) after 15 days of watering with non-saline water (Recovery). Yellow circles show the barycentres of the three halophyte species (*S. europaea*, *S. veneta*, *S. fruticosa*), orange triangles show the barycentres of the three experimental treatments (Ctrl, control; SS, salt stress (watering with 700 mM NaCl water solution); WS, water stress (complete withholding of irrigation)), and the light blue squares show the quantitative variables, i.e., the measured traits (fresh weight (FW), shoot water content (SWC), plant height (PH), number of branches (No.B), chlorophyll a (Chl. a), chlorophyll b (Chl. b), carotenoids (Caro), root sodium concentration (Na(r)), shoot sodium concentration (Na(s)), root chloride concentration (Cl(r)), shoot chloride concentration (Cl(s)), root potassium concentration (K(r)), shoot potassium concentration (K(s)), root calcium concentration (Ca(r)), shoot calcium concentration (Ca(s)), glycine betaine (GB), proline (PRO), total soluble sugars (TSS), malondialdehyde (MDA), hydrogen peroxide (H_2_O_2_), total phenolic compounds (TPC), total flavonoids (TF).

Two principal component analyses (PCAs) were performed to further evaluate the relationships among traits after the stress (PCAstress) and recovery (PCArecovery) treatments and to quantify the strength and direction of correlations between the original traits and the extrapolated principal components (PCs). The first three PCs (eigenvalues are reported in Table S1 of the Supplementary Materials) explained 62% and 53% of the total variance in PCAstress and PCArecovery, respectively, and were used for PCA interpretation. The correlation circles and the biplots of the first two components, PC1 and PC2, and the variables measured after the 30 days of stress (PCAstress) and the 15 days of recovery (PCArecovery) are reported in Figure 9.

In PCAstress, PC1 accounted for the differences between the salt stress treatment, whose barycentre was located on the positive side of PC1, and the water stress treatment, whose barycentre was located on the negative side of PC1 (Figure 9B). PC1 was positively correlated with Na(r) (0.87), Na(s) (0.85), Cl(s) (0.82), Cl(r) (0.79), Ca(r) (0.75), and FW (0.62), and negatively correlated with K(s) (−0.63) and PRO (−0.56) (Figure 9A), meaning that the accumulation of Na, Cl, and Ca is the primary mechanism helping to sustain plant growth under salt stress, whereas PRO production and K(s) accumulation are the main mechanisms adopted under water stress.

PC2 showed the relationship between Na^+^ and Cl^−^ accumulation, pigment production, and oxidative stress. PC2, indeed, presented the strongest positive correlations with Caro (0.84), Chl. a (0.83), Chl. b (0.75), and the highest negative correlations with Na(s) (−0.39) and Cl(s) (−0.39) (Figure 9A), meaning that the accumulation of these ions interfered with the production of pigments. Interestingly, the barycentres of the two annual species were located on the positive side of the PC2 axes, whereas the barycentre of *S. fruticosa* was located on the negative side (Figure 9B), indicating that pigment production was less affected by ion accumulation in this latter species.

PC3, finally, summarised the relationship between the plant species and the osmolytes. This third component was positively correlated with TSS (0.78), PRO (0.54), and TPC (0.42), and negatively correlated with GB (−0.51) (Table S2 of Supplementary Materials). *S. europaea* and *S. veneta* barycentres were placed on the positive side of the PC3 axis, whereas *S. fruticosa* was in the negative one (Table S3 of Supplementary Materials). This may suggest that the annual species rely on the production of sugars, proline, and phenolic compounds for osmotic adjustment under stress conditions, whereas the perennial species depends more on glycine betaine accumulation for its stress tolerance.

The PCArecovery outlined some evident changes: as in the PCAstress, the PC1 accounted for the different effects of the stress treatments, with the salt stress barycentre placed on the positive side of the PC1 axis and the water stress and control barycentres clustered on the negative side (Figure 9D), suggesting that, after recovery, water-stressed plants behaved similarly to control plants. PC1 was correlated positively with Na(r) (0.81), whose concentration decreased after recovery, especially in salt-treated plants, and negatively with K(r) (−0.55) (Figure 9C), whose concentration decreased after recovery, especially in the annual water-stressed plants.

The PC2 highlighted the differences between the annual *S. europaea* and the perennial *S: fruticosa*, with *S. veneta* showing an intermediate behaviour between the two other species. The barycentre of *S. europaea* was placed on the positive side of the PC2 axis (Figure 9D), which was positively correlated with PH (0.85), Caro (0.78), Chl. a (0.63), and Chl. b (0.44) (Figure 9C), whereas the barycentre of *S. fruticosa* was on the negative side. This placement reflects the fact that the recovery of these traits was more pronounced in *S. europaea* than in *S. fruticosa*, since these traits were compromised more seriously in the annual than in the perennial species under water stress.

Finally, the third component differentiated the control treatment, standing on the positive PC3 side (Table S3 of Supplementary Materials), from the water stress treatment, standing on the negative PC3 side. PC3 was positively correlated with Chl. a (0.64) and Chl. b (0.57), as control plants showed the highest pigment content even at the recovery stage and was negatively correlated to K(s) (−0.35) (Table S2 of Supplementary Materials), which increased in water-stressed plants after the recovery, especially in the two annual halophytes. 

## 3. Discussion

Cultivating drought- and salt-tolerant crops can build resilience to climate change and enhance farm productivity and livelihoods in drought- and salt-prone areas. Generally, salinity and drought regimes are not stable but fluctuate seasonally and geographically, depending on the climate and hydrological conditions of each specific environment. Thus, the extent to which a species can cope with these fluctuations is an important trait that can be selected for saline agriculture.

*Salicornia europaea*, *S. veneta*, and *Sarcocornia fruticosa* are three halophytic species already traded in the market as leafy vegetables and oil-seed crops, thanks to their high content of nutritional compounds with valuable health-related properties. The natural saline habitats of these species are especially sensitive to climate change effects, which will include more frequent, more intense, and longer drought periods and higher soil salinity levels, albeit with wide seasonal variations [44].

From a general overview of our results, all three species were shown to be remarkably tolerant to salinity but sensitive to water deficit, albeit to a lesser extent in *S. fruticosa*, which showed higher resistance to dehydration and greater ability to recover after drought exposition. Our findings are supported by the ecology and the evolutionary trends within this lineage of species. In the Mediterranean, the two genera grow in close sympatry but are separated ecologically [16]. *Salicornia* dominates inland or coastal lagoons which may remain flooded for longer periods after winter rains. By acquiring an annual life cycle, *Salicornia* species were able to adapt to more unstable habitats and to expand to colder northern areas [16]. European *Sarcorcornia* are frost sensitive and grow only in winter-mild Atlantic coasts or drier Mediterranean areas [14].

The surveyed *S. fruticosa* seeds were collected from a semiarid zone (La Albufera Natural Park, Valencia, Spain), with a mean annual temperature, precipitation, and evapotranspiration of 17.5 °C, 488 mm, and 1199 mm, respectively. On the other hand, the *S. europaea* and *S. veneta* seeds were sampled from a more humid area (Piallassa della Baiona, Ravenna, Italy), having mean annual temperature, precipitation, and evapotranspiration of 14.6 °C, 576 mm, and 828 mm, respectively. This difference in environmental conditions may be the primary reason for developing a more robust drought tolerance in *S. fruticosa*. However, the slower metabolism of perennial plants could represent an advantageous adaptive strategy for survival under stress conditions since it allows for the saving of water and resource consumption while enhancing the synthesis of protective compounds [45]. This may have contributed to the better performance of the perennial *S. fruticosa* under water stress with respect to the annual *S. europaea* and *S. veneta.*

Photosynthetic pigment contents in *S. fruticosa* were not affected by salinity or drought stress, whereas a reduction in pigment contents was recorded in *S. europaea* and *S. veneta*, being generally modest under salt stress but severe in response to water deficit. Here again, these differences could be a consequence of the better adaptation of *S. fruticosa* to semiarid conditions or dependent on its life cycle type. When exposed to stress, annual plants hasten the transition from the vegetative to the reproductive stage, activating a process of stress-induced senescence that shifts nutrient allocation to developing seeds [46,47]. The stress-induced senescence is regulated differently and occurs more gradually in the perennial plants, since they can also propagate vegetatively. When they experience stress, perennial plants prioritise biomass accumulation in roots, whose contribution to stress avoidance is fundamental, protect photosynthetic tissues to sustain C assimilation and boost the source strength, and enhance the conservation of meristematic tissues, which are essential for recovering after the stress period [48,49]. This basic distinction may also explain the different variations in pigment contents under stressful conditions between the perennial *S. fruticosa* and the two annual *S. europaea* and *S. veneta*. In any case, the two annual species were able to restore their pigment pools during the recovery phase.

Similar ion accumulation patterns were observed in all three species, with a consistent increase in Na^+^ and Cl^−^ concentrations at the root and shoot level in response to high salinity. This response is in line with the finding that halophytes can take up and efficiently compartmentalise the ions naturally present in the growth media to conserve the water potential gradient and maintain water uptake [50]. The salt-treated plants retained their high content of Na^+^ and Cl^−^ in the shoots notwithstanding the recovery treatment, since the transport of these ions, to be used as inorganic osmolytes, is energetically cheaper than the *de novo* synthesis of organic osmolytes [51]. It should also be pointed out that Na^+^ and Cl^−^ content in shoots were very high, and much higher than in roots, in the absence of salt; that is, in the control and water stress treatments. This result indicates the active transport of these ions to the aboveground organs, even at low external salinity, so that Na^+^ and Cl^−^ can contribute to cellular osmotic balance also in non-stressed and water-stressed plants.

Salinity, however, caused a decrease in K^+^ translocation to the shoots, likely related to the antagonism between K^+^ and Na^+^ ions, which are physicochemically similar [52]. This is evident in the PCAstress correlation circle, where the Na and Cl arrows are opposite to the K(s) arrow, implying that an increase in the former ions caused a decrease in the latter ion. The significant increase in K^+^ shoot allocation under water stress suggested that this ion is a key osmoticum used to maintain water status in *Salicornia* and *Sarcocornia* spp. under water stress conditions. Indeed, water-stressed plants held a high K^+^ shoot content even after recovery.

The significant increase in Ca^2+^ concentration under high salinity conditions in both below- and aboveground organs supports the notion that Ca, being involved in a diverse array of sensor proteins, plays a central role in orchestrating the whole-plant response to salt stress [53,54]. Indeed, Ca^2+^ content was positively correlated with Na^+^ and Cl^−^ contents in the PCAstress correlation circle. The ability to preserve Ca uptake and retention under salinity seems to be a common feature of halophytes, since it was also reported in other salt-tolerant species such as *Sarcobatus vermiculatus*, *Climacoptera turcomanica*, *Salicornia persica*, *Halimocnemis pilifera*, *Petrosimonia glauca*, and *Atriplex verrucifera* [55].

To sum up, the effects of recovery on ion contents were relevant on roots, which are the organs more directly and dynamically in contact with the external environment, whereas ion remobilisation within shoots was not substantially affected by the recovery treatment.

Besides accumulating inorganic ions, glassworts species synthesise several organic osmolytes under osmotic stress, which contribute to cellular osmotic adjustment, free radical scavenging, and the activation of specific signalling pathways.

In both the stressed and non-stressed plants of the two genera, *Salicornia* and *Sarcocornia*, relatively high absolute values of GB were quantified, suggesting that GB accumulation is a constitutive defence mechanism against osmotic stress. Responses of these plants to abiotic stress probably rely more on changes in GB subcellular compartmentalisation, i.e., GB redistribution from the vacuole to the cytoplasm, rather than its de novo synthesis. There is indeed evidence for stress-induced changes in the intracellular localisation of compatible solutes in halophytes, for example, in *Limonium latifolium* [56]; however, data on these putative mechanisms are still scarce. Still, GB concentration can increase in response to stress, as observed under salinity in *S. veneta* and, mostly, in water-stressed *S. fruticosa* plants, suggesting that the higher drought tolerance of this latter species is partly due to a relatively higher GB accumulation.

Proline (PRO) is probably the most common compatible solute in plant species [57]. Nevertheless, no significant change in PRO concentration was detected in our experiments, except for the increase under water stress in *S. europaea* and *S. veneta*. However, the measured absolute PRO concentrations were too low to have any relevant osmotic effect when compared to GB or ion contents in the shoots. Still, PRO could have contributed to enhanced stress tolerance through its additional ability to scavenge ROS, directly stabilise proteins and other cellular structures, and provide cellular redox potential [58].

Comparing these outcomes, it appears that GB is the major organic osmolyte contributing to drought tolerance in *S. fruticosa*, whereas PRO plays a relatively more relevant role *in S. europaea* and *S. veneta*. Indeed, after recovery from water stress, a drop in GB concentration was observed in *S. fruticosa*, and PRO levels decreased significantly in *S. europaea* and *S. veneta*. These results are in agreement with the findings reported by Gil et al. [41], who measured high (>400 μmol g^−1^ DW) GB and very low (1–2 μmol g^−1^ DW) PRO concentrations in *S. fruticosa* under field conditions in the aforementioned semiarid La Albufera Natural Park, and with the results of Parida and Jha [59], who found PRO to be the main organic osmolyte accumulated in response to drought stress in *Salicornia brachiata*.

This supports the assumption that typical GB-accumulating species generally contain low PRO levels and vice versa [60], as already observed in many species, including both halophytes and glycophytes. For example, in the halophyte *Spartina alternifolia*, in the presence of 600 mM NaCl, GB contents were 10-fold higher than those of PRO (ca. 150 vs. 15 μmol g^−1^ FW, respectively) [61]. The differences were much more pronounced in another halophyte, *Halocnemum strobilaceum*, showing GB values > 200-fold greater than those of PRO (700 vs. 3 μmol g^−1^ DW) under 690 mM NaCl [62]. A similar pattern, although with much lower absolute values, was found in the glycophyte *Spinacia oleracea* in the presence of 170 mM NaCl, showing GB concentrations (3.25 μmol g^−1^ FW) about four-fold higher than those of PRO (0.78 μmol g^−1^ FW) [63]. Conversely, PRO appears to contribute relatively more to osmotic balance under drought conditions (200–400 μmol g^−1^ DW) than GB (40–60 μmol g^−1^ DW) in the genus *Capsicum* [64]. The halophyte *Juncus maritimus* also accumulated PRO rather than GB in response to salt stress (400 mM NaCl): ca. 130 vs. 25 μmol g^−1^ DW, respectively [65]. Similarly, a preferential accumulation of PRO over GB was observed in the halophyte *Limonium santapolense* under drought stress (ca. 120 vs. 23 μmol g^−1^ DW, respectively) [66].

The accumulation of the total soluble sugars (TSS) may enhance drought tolerance in *S. europaea*, since TSS levels increased in response to the water stress treatment; however, their contribution to *S. veneta* and *S. fruticosa* stress resistance was negligible. This result is in contrast to previous studies that have reported TSS accumulation as the primary mechanism for osmotic adjustment in *S. fruticosa* [20] and *Salicornia persica* [67]. However, as discussed by Gil et al. [68], sugar accumulation should be interpreted with caution. In fact, unlike other osmolytes occurring in plants at very low levels, unless stressful conditions stimulate their biosynthesis, soluble sugars are components of primary metabolism that play different functional roles unrelated to stress responses. This may be the reason why no significant changes in TSS contents were observed after stress recovery in any of the three studied species.

The fact that the stress treatments did not increase the levels of oxidative stress markers, i.e., MDA and H_2_O_2_, revealed that no oxidative stress was generated by salt or water stress in any of the three species. In some cases—salt stress in *S. europaea* and water stress in *S. veneta*—the contents of the oxidative stress markers, i.e., MDA and H_2_O_2_, even decreased with respect to the non-stressed controls. This response may be due to the increased activity of peroxidase, which is generally stored in the peroxisome and vacuoles, and plays an active role in reducing oxidative stress decreasing lipid peroxidation [69].

Consequently, we did not detect a significant accumulation of non-enzymatic, antioxidant compounds, i.e., total phenolic (TPC) or flavonoid (TF) compounds. This is reflected in the PCAstress correlation circle, in which the short and faded MDA, H_2_O_2_, TPC, and TF arrows denote a weak contribution of these traits to the variability of the whole dataset.

Taken together, these results suggest that the stress responses based on ion transport control and osmolyte accumulation were efficient enough to avoid or even reduce oxidative stress under our experimental conditions. However, we must note that the absence of oxidative stress may also result, at least in part, from efficient enzymatic ROS-detoxifying machinery, based on the activity of antioxidant enzymes such as superoxide dismutase, catalase, ascorbate peroxidase, glutathione peroxidase, and peroxiredoxin [70], among others, which were not specifically addressed in this study.

## 4. Materials and Methods

### 4.1. Plant Material

Seeds of *Salicornia europaea* and *Salicornia veneta* were collected from Pialassa della Baiona, a coastal lagoon located within the Po Delta Regional Park in Italy. Seeds of *Sarcocornia fruticosa* were collected from ‘La Albufera’ Natural Park, located near the city of Valencia, Eastern Spain. Mean annual values of climatic parameters from 2006 to 2021 in the two sampling areas are reported in Table 2. The experiments were carried out in the laboratories and greenhouses of the Institute for the Conservation and Improvement of Valencian Agrodiversity (COMAV), Polytechnic University of Valencia, Spain.

Seeds were sown manually in plastic trays filled with commercial peat, placed into a growth chamber with a 16/8-h light/dark cycle, day/night temperatures of 25/22 °C, and 70–80% relative humidity and watered thrice per week with tap water. Forty days after sowing, seedlings of each species of uniform size and shape were transplanted into plastic pots (12 cm diameter) filled with 500 g of a mix of commercial peat (26% organic carbon, pH_H_2___O_ = 7.0, and EC = 0.6 dS m^−^^1^) and perlite (80:20 *v*/*v*). Three seedlings were transplanted to each pot. The pots were transferred into the controlled environment of a greenhouse, placed over benches, and irrigated manually with tap water thrice per week. During the experimental period in the greenhouse, temperatures ranged between 21.3 ± 1.6 and 28.6 ± 1.8 °C and RH between 67.5 ± 9.9 and 92.6 ± 2.9%.

### 4.2. Experimental Design and Stress Treatments

Four weeks after transplanting, when the plantlets were fully established, the pots with individuals of each species were randomly divided into three groups and subjected to the following treatments: control (Ctrl, irrigation with tap water thrice per week), salt stress (SS, irrigation with a 700 mM NaCl aqueous solution, thrice per week), and water stress (WS, complete withholding of irrigation). Pots were placed in trays and were watered from the bottom, i.e., filling the trays, considering a volume of 0.13 L pot^−1^. After one month of treatment, the stressed plants were allowed to recover during the following fifteen days through intensive pot washings with tap water in the salt stress treatment and through the restoring of the soil moisture level up to 80% in the drought-stress treatment. In this phase, pots were watered from the top (0.13 L pot^−1^ for Ctrl and 0.50 L pot^−1^ for SS and WS) and, only in the SS treatments, the drainage water was always discarded to remove the leached salt. The amount of water (L pot^−1^) distributed per each treatment during the Stress and Recovery phases are shown in Table 3.

The three factors, plant species (PS, 3 levels), stress treatments (ST, 3 levels), and harvesting time (HT, 2 levels), were cross-combined, resulting in 18 treatments. Four completely randomised replicates were set up, totalling 72 pots. This number of replicates is quite commonly adopted in pot experiments on this topic [29,74,75,76].

The plants were harvested twice, the first half after the thirty days of stress treatments (T30) and the second half after the fifteen days of recovery (T45). Morphological parameters were determined on all individual plants (*n* = 12 per species and treatment). Samples of the aboveground biomass, i.e., of the leafless succulent green stems, were used for biochemical analysis; in this case, the shoots of the three plants grown in each pot were pooled (*n* = 4 per species and treatment, but each sample was a pool of three independent plants).

### 4.3. Plant Growth

**Figure 10:**
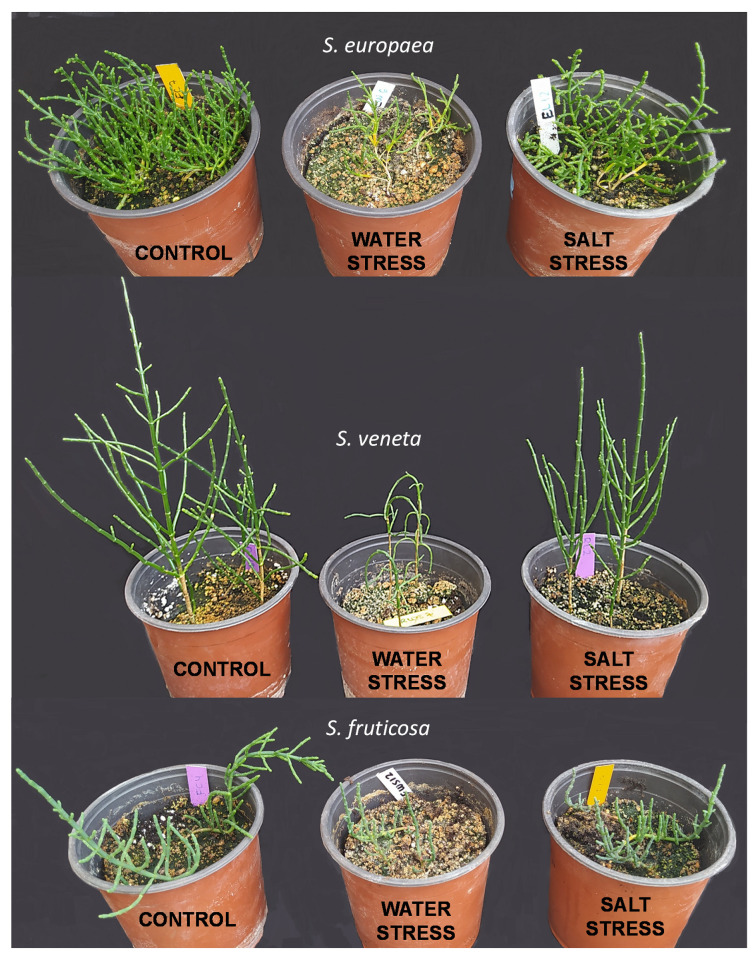
Picture of the three halophytes species after thirty days of stress treatments: control; water stress (complete withholding of irrigation); salt stress (watering with 700 mM NaCl).

The three surveyed species are characterised by strongly reduced leaves, which are embedded to form articulated, photosynthetically active succulent stems appearing to be composed of jointed segments (Figure 10). The number of branches (excluding the main branch) and plant height were determined at the beginning of the treatments (T0), after fifteen (T15) or thirty (T30) days of the stress treatments and after 15 days of recovery; that is, 45 days from the beginning of the experiment (T45). At both harvests, ‘Stress’ and ‘Recovery’, the aboveground biomass of each plant was separated from the root and weighed (fresh weight, FW). Roots were cleaned with a brush and weighed. Portions of the shoots and the root material were oven-dried at 65 °C until a constant weight was reached (ca. 72 h) and were then weighed again (dry weight, DW) to determine the water content percentage according to the following formula:

(1) $$\mathrm{WC}\;\left(\%\right)=\frac{\mathrm{FW}-\mathrm{DW}}{\mathrm{FW}}\times 100$$

Fresh shoot material was flash-frozen in liquid N_2_ and stored at −75 °C, and dry material was stored at room temperature in tightly closed paper envelopes. Pot substrate was collected at each harvest time to determine moisture and electrical conductivity (EC) in the laboratory. Substrate moisture was calculated gravimetrically, as described above for the plant samples (Equation (1)). For EC measurements, a 1:5 suspension of the dry substrate and deionised water was prepared and mixed for one hour at 600 rpm and 21 °C before being filtered. The EC was measured with a Crison 522 conductivity meter and expressed in dS m^−1^.

### 4.4. Photosynthetic Pigments

The concentrations (mg g^−1^ DW) of chlorophyll a (Chl. a), chlorophyll b (Chl. b), and carotenoids (Caro) in the plant tissues were measured spectrophotometrically, according to a previously described method [77]. Fresh ground shoot material (ca. 0.05 g) was extracted with 1 mL of ice-cold 80% acetone. The samples were mixed during 12 h in a shaker in the dark and then centrifuged at 13,300× *g* for 10 min at 4 °C. The supernatant absorbance was measured at 470, 646, and 663 nm, and the pigment concentrations were calculated, applying the equations described by Lichtenthaler and Wellburn [77].

### 4.5. Ion Quantification

The concentrations of Na^+^, Cl^−^, K^+^, and Ca^2+^ were calculated separately for roots and shoots following the procedure described by Weimberg [78]. Two mL of Milli-Q water were added to ca 0.1 g of dry plant material, vortexed, and then mixed for 24 h in a shaker. The samples were then incubated in a water bath for 30 min at 95 °C, cooled on ice, and filtered through a 0.45 μm nylon filter. The cations were quantified with a PFP7 flame photometer (Jenway Inc., Burlington, VT, USA), whereas the anions were measured using a chlorimeter (Sherwood, model 926, Cambridge, UK).

### 4.6. Quantification of Osmolytes

The concentration of glycine betaine (GB) was determined as described by Grieve and Grattan [79], with some modifications [80]. Fresh shoot material (0.15 g) was shaken for 24 h at 4 °C with 1.5 mL Mili Q water and then centrifuged at 13,300× *g* for 10 min. The supernatant was mixed (1:1) with a 2N H_2_SO_4_ solution and stored in ice for 1 h. Then, 125 μL of the sample were supplemented with 50 μL of ice-cold KI-I_2_ solution, which induces glycine betaine precipitation in the form of golden crystals. All the following steps were completed in the dark. The samples were maintained at 4 °C for 16 h and then centrifuged at 13,300× *g* for 45 min at 0 °C. The supernatant was carefully removed, and the glycine betaine crystals were dissolved into 1.4 mL of cold 1,2-dichloroethane; the tubes were kept for 2.5 h under dark and cold conditions, and, finally, their absorbance was recorded at 365 nm. Glycine betaine concentration was calculated against a GB standard calibration curve and expressed as μmol g^−1^ DW.

Proline (PRO) was quantified following the protocol of Bates et al. [81]. Fresh aboveground material (ca. 0.05 g) was extracted in 3% (*w*/*v*) aqueous sulpho-salicylic acid and subsequently supplemented with acid ninhydrin, incubated in a water bath for 1 h at 95 °C, cooled on ice, and then extracted with two volumes of toluene. The absorbance of the organic phase was read with a spectrophotometer at 520 nm, using toluene as a blank. A standard curve was obtained by running parallel assays with known PRO amounts. PRO concentration was expressed as μmol g^−1^ DW.

Total soluble sugars (TSS) were measured from ca. 0.05 g of ground fresh material extracted with 2 mL 80% (*v*/*v*) methanol, according to the method described by Dubois et al. [82]. After mixing in a shaker for 24 h, the samples were centrifuged at 13,300× *g* for 10 min; the supernatants, appropriately diluted with water, were mixed with 95% sulphuric acid and 5% phenol. After 20 min incubation at room temperature, the absorbance was measured at 490 nm. TSS concentration was expressed as equivalents of glucose, used as the standard (mg eq. glucose g^−1^ DW).

### 4.7. Determination of Oxidative Stress Markers and Antioxidant Compounds

Malondialdehyde (MDA), total phenolic compounds (TPC), and total flavonoids (TF) were quantified in the same methanol extracts prepared for TSS measurements.

The method defined by Hodges et al. [83] was used for MDA quantification, with some modifications [84]. Extracts were mixed with 0.5% thiobarbituric acid (TBA) prepared in 20% trichloroacetic acid (TCA)—or with 20% TCA without TBA for the controls—and then incubated at 95 °C for 20 min, cooled on ice, and centrifuged at 13,300× *g* for 10 min at 4 °C. The supernatant absorbance was measured at 532 nm. The non-specific absorbance at 600 and 440 nm was subtracted, and the MDA concentration was computed, applying the equations proposed by Taulavuori et al. [84]. MDA contents were expressed as nmol g^−1^ DW.

Hydrogen peroxide content in plants was quantified as previously described [85]. Fresh plant material (0.05 g) was extracted with a 0.1% (*w*/*v*) trichloroacetic acid (TCA) solution. After centrifugation, the supernatant was mixed with one volume of 10 mM potassium phosphate buffer (pH 7.0) and two volumes of 1 M potassium iodide. The absorbance of the samples was determined at 390 nm. Reaction mixtures containing known concentrations of H_2_O_2_ were assayed in parallel to obtain a standard curve, and H_2_O_2_ concentrations were expressed as μmol g^−1^ DW.

TPC were measured by reaction with the Folin–Ciocalteu reagent, following the method previously [86]. The methanol extracts were mixed with Na_2_CO_3_, incubated at room temperature in the dark for 90 min, and the absorbance was read at 765 nm. Gallic acid (GA) was used as standard, and the measured TPC concentrations were expressed as GA equivalents (mg eq. GA g^−1^ DW).

TF were quantified by a previously described protocol [87], namely by sample incubation with NaNO_2_, followed by a reaction with AlCl_3_. After the reaction, the sample absorbance was determined at 510 nm, and TF contents were expressed as equivalents of the catechin standard (mg eq. C g^−1^ DW).

### 4.8. Statistical Analysis

The data of the measured traits within each plant species (PS) were subjected to two separated one-way ANOVAs for the respective stress treatments (ST) and harvesting times (HT). The Tukey’s honestly significant difference (HSD) post hoc test at *p* < 0.05 was applied to indicate significant differences among levels in significant ANOVA sources. A two-way ANOVA was then performed to assess the interaction between stress treatment (ST) and harvesting time (HT). The two-way ANOVA results are reported in Table S4 of Supplementary Materials.

We investigated the relationships between the 22 traits measured within each halophyte species by computing the Pearson correlation coefficients (r) and then testing their significance with α = 0.05. For each species, the correlation matrix is shown as a network diagram where each entity of the dataset represents a node, and highly correlated variables are clustered together. Each path represents a correlation between the two variables it joins. A blue path represents a positive correlation, and a red path represents a negative correlation. Only significant correlations (*p* < 0.05) are represented. The width and transparency of the line represent the strength of the correlation (wider and less transparent = stronger correlation).

Two principal component analyses were carried out on the data collected at the first (PCAstress) and second harvest time (PCArecovery) to summarise the performances outlined by the three genotypes under the Stress and Recovery periods with a multivariate approach.

The principal components (PCs) were obtained from centred and scaled quantitative variables through the diagonalisation of the correlation matrix and extraction of the associated eigenvectors and eigenvalues. All 22 measured traits were set as active quantitative variables, whereas the three halophyte species (*S. europaea*, *S. veneta*, and *S. fruticosa*) and the three treatments (Ctrl, SS, WS) were used as supplementary categorical variables, i.e., variables that were not used in the computation of PCs. The Pearson correlation coefficients were determined between the PCs and each quantitative variable (the 22 measured traits). The associated *p*-values were calculated to classify the variables according to their relevance (Table S2 of Supplementary Materials).

All the statistical analyses were performed with the R 6.3.6 statistical software, using Car [88] and Emmeans [89] packages for the analysis of variance and post hoc test, and the FactoMineR package for principal component analysis [90]. Charts were created with the ggplot2 [91] and corrr [92] R packages.

## 5. Conclusions

The three investigated halophytes, the annual *S. europaea* and *S. veneta* and the perennial *S. fruticosa*, are highly tolerant to salinity but sensitive to water stress, although the latter species to a lesser extent. Salt tolerance seems to depend mainly on the salt-induced accumulation of ions (Na^+^, Cl^−^ and Ca^2+^) and the shoot biosynthesis of organic osmolytes, both contributing to osmotic adjustment under stress. Active transport of these ions to the aerial part of the plants and high concentrations of glycine betaine have also been detected in the control, non-stressed plants, indicating that these defence mechanisms against stress are at least partially constitutive.

The higher drought tolerance of *S. fruticosa*, compared to its annual counterparts, was reflected in a relatively lower reduction in shoot fresh weight and the absence of a decrease in photosynthetic pigment content under water deficit conditions and was attributed to the relatively higher accumulation of glycine betaine. *Sarcocornia fruticosa* also showed total recovery capacity after the water stress treatment, whereas the fresh weight of the water-stressed plants of *S. europaea* and *S. veneta* remained at values significantly lower than the controls after the recovery period.

Neither salinity nor drought stress generated oxidative stress. Consequently, the presence of stress response mechanisms based on the activation of antioxidant systems was not expected; indeed, no significant increase in the levels of antioxidant compounds was detected in any of the three halophytes. However, further studies should be carried out to assess the possible contribution of enzymatic antioxidant activities to the whole antioxidant network of these species.

The higher drought tolerance observed in *S. fruticosa* with respect to the two *Salicornia* species could be based on differences in the environmental conditions of the plants’ natural habitats, as it is drier for *S. fruticosa*. However, a more gradual process of stress-induced senescence in the perennial *S. fruticosa* compared to the annual *S. europaea* and *S. veneta*, might have allowed water-stressed plants to preserve their pool of photosynthetic pigments and recover to control fresh weight after rewatering. Further studies will be required to confirm this hypothesis, including, for instance, the assessment of the responses to water deficit of annual and perennial plants growing in the same natural habitat.

## Figures and Tables

**Table 1:** Plant height (cm) and number of branches in the three halophytes (SE, *S. europaea*; SV, *S. veneta*; SF, *S. fruticosa*) measured at the beginning (T0) and after 15 (T15), 30 (T30), or 45 (T45) days of starting the stress treatments. Ctrl, control; SS, salt stress (watering with 700 mM NaCl); WS, water stress (complete withholding of irrigation). The values shown are means ± SE (*n* = 4). For each species, different lowercase letters in a column indicate significant differences between the three treatments within the same sampling time, whereas different uppercase letters in each row indicate significant differences between sampling times for the same treatment, at *p* ≤ 0.05.

		Plant Height (PH) (cm)				Number of Branches (No. B)			
		T0	Stress (T15)	Stress (T30)	Recovery (T45)	T0	Stress (T15)	Stress (T30)	Recovery (T45)
**SE**	**Ctrl**	5.8 ± 0.3 aC	8.7 ± 0.5 aB	12.9 ± 1.0 aA	12.3 ± 1.0 aA	5.7 ± 0.5 aC	10.2 ± 0.7 aB	18.1 ± 1.8 aA	23.8 ± 3.3 aA
	**SS**	5.7 ± 0.3 aC	8.5 ± 0.4 aB	11.4 ± 0.5 aA	10.5 ± 0.6 aA	6.3 ± 0.5 aC	11.0 ± 0.7 aB	18.2 ± 1.2 aA	20.8 ± 1.5 aA
	**WS**	5.3 ± 0.3 aC	7.6 ± 0.4 aB	7.0 ± 0.6 bA	10.4 ± 0.9 aA	6.0 ± 0.5 aB	12.0 ± 2.1 aAB	10.3 ± 1.5 bA	16.9 ± 2.4 aA
**SV**	**Ctrl**	9.8 ± 0.4 aC	14.7 ± 0.7 aB	21.6 ± 2.0 aA	22.6 ± 1.8 aA	2.1 ± 0.3 aC	6.9 ± 0.6 aB	11.8 ± 1.6 aA	13.0 ± 1.7 aA
	**SS**	10.2 ± 0.4 aC	15.8 ± 0.5 aB	20.6 ± 0.8 abA	19.9 ± 1.5 aA	1.5 ± 0.3 aC	8.0 ± 0.6 aB	10.1 ± 0.9 aA	15.0 ± 3.4 aA
	**WS**	9.6 ± 0.5 aC	15.1 ± 0.5 aB	16.1 ± 0.6 bA	19.6 ± 1.5 aA	1.5 ± 0.3 aC	7.3 ± 0.5 aB	9.5 ± 0.8 aB	9.3 ± 2.4 aA
**SF**	**Ctrl**	5.6 ± 0.3 aC	8.9 ± 0.5 aB	13.1 ± 1.3 aA	14.6 ± 1.3 aA	0.4 ± 0.2 aD	8.1 ± 1.0 aC	18.9 ± 3.0 aB	28.5 ± 1.5 aA
	**SS**	5.1 ± 0.4 aC	9.2 ± 0.4 aB	11.6 ± 0.7 aA	13.1 ± 0.6 aA	0.4 ± 0.2 aC	8.8 ± 1.0 aB	18.4 ± 2.3 aA	21.7 ± 1.7 bA
	**WS**	5.0 ± 0.3 aC	8.3 ± 0.5 aB	10.0 ± 0.9 aA	12.6 ± 0.7 aA	0.5 ± 0.2 aD	8.7 ± 1.0 aC	14.0 ± 2.4 aB	20.8 ± 2.5 bA

**Table 2:** Historical weather data (from 2006 to 2021) of the areas of ‘La Albufera’ Natural Park (Spain) and Piallassa della Baiona (Italy), provided, respectively, by the Spanish Agroclimatic Information System for Irrigation (SIAR) and the Italian Arpae-Simc meteorological network [71,72]. T: temperature; RH: relative humidity; Eto: evapotranspiration. Eto data of Piallassa della Baiona were calculated applying the Thornthwaite method [73].

	‘La Albufera’ Natural Park				Piallassa Della Baiona			
Year	Mean T	Mean RH	Rainfall	ET_0_	Mean T	Mean RH	Rainfall	ET_0_
	(°C)	(%)	(mm)	(mm)	(°C)	(%)	(mm)	(mm)
2006	17.53	69.13	464.40	1189.38	14.40	77.64	337.65	814.71
2007	16.81	68.13	894.40	1164.50	14.20	73.18	490.00	809.25
2008	16.88	68.35	674.40	1194.10	14.20	73.63	491.13	804.14
2009	17.34	68.60	446.20	1215.26	14.19	72.79	555.86	816.07
2010	16.78	68.31	565.00	1206.22	13.23	74.09	450.00	776.35
2011	17.57	70.32	472.00	1166.73	14.76	71.36	346.60	846.35
2012	17.31	67.58	503.61	1208.25	14.71	69.98	563.60	864.97
2013	17.55	63.26	263.80	1245.42	14.49	72.86	870.20	822.93
2014	18.32	65.32	224.40	1278.22	15.60	73.91	740.00	833.27
2015	17.76	70.02	401.26	1169.08	15.20	77.18	616.80	860.61
2016	17.85	68.66	259.57	1218.41	14.71	80.86	829.40	825.33
2017	17.59	68.51	307.26	1238.82	14.84	76.69	641.80	851.52
2018	17.60	68.06	684.02	1225.71	15.32	78.53	613.60	870.93
2019	17.79	66.59	427.00	1243.83	15.03	81.94	780.80	839.65
2020	18.09	72.95	731.94	1186.44	14.70	76.76	556.40	808.83
2021	17.50	75.40	494.72	1039.10	14.45	75.75	335.00	809.89
*Mean*	*17.52*	*68.70*	*488.37*	*1199.34*	*14.63*	*75.45*	*576.18*	*828.42*

**Table 3:** Amount of water distributed per pot during the stress period (Stress) and the recovery period (Recovery) in the three treatments (Ctrl, control; SS, irrigation with 700 mM NaCl; WS, complete withholding of irrigation).

	Stress (L pot^−1^)	Recovery (L pot^−1^)	Total (L pot^−1^)
**Ctrl**	1.75	1	2.75
**SS**	1.75	4	5.75
**WS**	0	2	2

## Data Availability

Data is contained within the article or supplementary material.

## Supplementary Materials

The following are available online at https://www.mdpi.com/article/10.3390/plants11081058/s1. Table S1. Eigen analysis of PCAstress and PCArecovery correlation matrix; Table S2. Correlation coefficients between the first three PCs (PC1, PC2, PC3) and the quantitative variables traits (fresh weight (FW), shoot water content (SWC), plant height (PH), number of branches (No.B), chlorophyll a (Chl. a), chlorophyll b (Chl. b), carotenoids (Caro), root sodium concentration (Na(r)), shoot sodium concentration (Na(s)), root chloride concentration (Cl(r)), shoot chloride concentration (Cl(s)), root potassium concentration (K(r)), shoot potassium concentration (K(s)), root calcium concentration (Ca(r)), shoot calcium concentration (Ca(s)), glycine betaine (GB), proline (PRO), total soluble sugars (TSS), malondialdehyde (MDA), hydrogen peroxide (H_2_O_2_), total phenolic compounds (TPC), total flavonoids (TF). The PCs were computed using 22 input data. Significance codes: ^ns^, ^(+)^, *, **, and *** mean, respectively, not significant and significant at *p* ≤ 0.1, *p* ≤ 0.05, *p* ≤ 0.01 and *p* ≤ 0.001; Table S3. Coordinates of the barycentres of the supplementary categorical variables in PCAstress and PCArecovery biplots, respectively; Table S4. Two-way analysis of variance (ANOVA) of stress treatments (ST), harvesting time (HT), and their interactions (STxHT) for the three halophyte species, for the 22 measured traits (fresh weight (FW), shoot water content (SWC), plant height (PH), number of branches (No.B), chlorophyll a (Chl. a), chlorophyll b (Chl. b), carotenoids (Caro), root sodium concentration (Na(r)), shoot sodium concentration (Na(s)), root chloride concentration (Cl(r)), shoot chloride concentration (Cl(s)), root potassium concentration (K(r)), shoot potassium concentration (K(s)), root calcium concentration (Ca(r)), shoot calcium concentration (Ca(s)), glycine betaine (GB), proline (PRO), total soluble sugars (TSS), malondialdehyde (MDA), hydrogen peroxide (H_2_O_2_), total phenolic compounds (TPC), total flavonoids (TF). Significance codes: ^ns^, ^(+)^, *, **, and *** mean, respectively, not significant and significant at *p* ≤ 0.1, *p* ≤0.05, *p* ≤ 0.01, and *p* ≤ 0.001.
