# Supplementary material for: Recovery from Salinity and Drought Stress in the Perennial Sarcocornia fruticosa vs. the Annual Salicornia europaea and S. veneta
Source: Plants (Basel). 2022 Apr 13;11(8):1058. doi: 10.3390/plants11081058 (PMC9031041; doi:10.3390/plants11081058)
Supplement: Supplementary file 1 [file plants-11-01058-s001.zip › Supplementary_Plants 2022_Calone et al.pdf]

Table S1: Eigen analysis of PCAstress and PCArecovery correlation matrix

		Eigenvalue	Percentage of variance	Cumulative percentage of variance
PCAstress	PC1	5.68	25.83	25.83
	PC2	5.18	23.56	49.39
	PC3	2.85	12.97	62.36
PCArecovery	PC1	5.46	24.83	24.83
	PC2	3.80	17.28	42.11
	PC3	2.32	10.55	52.67

Table S2. Correlation coefficients between the first three PCs (PC1, PC2, PC3) and the quantitative variables traits (fresh weight (FW), shoot water content (SWC), plant height (PH), number of branches (No.B), chlorophyll a (Chl.a), chlorophyll b (Chl.b), carotenoids (Caro), root sodium concentration (Na(r)), shoot sodium concentration (Na(s)), root chloride concentration (Cl(r)), shoot chloride concentration (Cl(s)), root potassium concentration (K(r)), shoot potassium concentration (K(s)), root calcium concentration (Ca(r)), shoot calcium concentration (Ca(s)), glycine betaine (GB), proline (PRO), total soluble sugars (TSS), malondialdehyde (MDA), hydrogen peroxide (H_2O_2), total phenolic compounds (TPC), total flavonoids (TF). The PCs were computed using 22 input data. Significance codes: ^{ns}, $^{(+)}$, *, **, and *** mean, respectively, not significant and significant at $p \leq 0.1$, $p \leq 0.05$, $p \leq 0.01$ and $p \leq 0.001$

	PCAstress			PCArecovery		
	PC1	PC2	PC3	PC1	PC2	PC3
FW	0.62***	0.56***	0.01 ns	0.67***	0.06 ns	0.37*
SWC	0.51**	0.43**	-0.40*	0.62***	-0.07 ns	0.29(+)
PH	0.20 ns	0.57***	0.02 ns	0.20 ns	0.84***	0.01 ns
No.B	-0.24 ns	0.02 ns	-0.54***	-0.34*	-0.44**	0.33(+)
Chl.a	0.29 ns	0.83***	-0.15 ns	-0.10 ns	0.63***	0.64***
Chl.b	0.57***	0.75***	0.01 ns	0.22 ns	0.44**	0.57***
Caro	0.32(+)	0.84***	0.03 ns	0.22 ns	0.78***	0.37*
Na(r)	0.87***	-0.34*	0.16 ns	0.81***	-0.13 ns	0.01 ns
Na(s)	0.85***	-0.39*	0.00 ns	0.78***	-0.42*	0.06 ns
Cl(r)	0.79***	-0.37*	0.25 ns	0.22 ns	0.16 ns	-0.22 ns
Cl(s)	0.82***	-0.39*	0.03 ns	0.68***	-0.51**	0.23 ns
K(r)	-0.07 ns	0.46**	0.20 ns	-0.55***	0.01 ns	0.42*
K(s)	-0.63***	0.28 ns	0.43**	-0.36*	0.61***	-0.35*
Ca(r)	0.75***	-0.29(+)	0.33*	0.75***	-0.18 ns	-0.27 ns
Ca(s)	0.49**	-0.26 ns	0.42*	0.79***	0.23 ns	-0.14 ns
GB	0.02 ns	-0.35*	-0.51**	-0.06 ns	-0.52**	0.38*
PRO	-0.56***	-0.20 ns	0.54***	-0.27 ns	-0.48**	0.26 ns
TSS	-0.20 ns	-0.03 ns	0.78***	0.44**	0.05 ns	-0.12 ns
MDA	0.18 ns	0.51**	0.52**	0.77***	0.07 ns	0.11 ns
H_2O_2	-0.05 ns	0.69***	-0.25 ns	0.02*	0.26 ns	0.37*
TPC	-0.23 ns	0.11 ns	0.42*	0.32(+)	0.44**	-0.49**
TF	0.07ns	0.70***	0.33*	0.07 ns	0.06 ns	0.12 ns

Table S3. Coordinates of the barycentres of the supplementary categorical variables in PCAstress and PCArecovery biplots, respectively.

	Supplementary categorical variables	PC1	PC2	PC3
PCAstress	*S. europaea*	0.059	0.291	0.893
	S. veneta	0.434	0.936	0.760
	S. fruticosa	-0.493	-1.227	-1.653
	Ctrl	-0.416	2.479	-0.600
	SS	2.892	-1.488	0.232
	WS	-2.476	-0.990	0.368
PCArecovery	*S. europaea*	0.972	1.975	-0.396
	S. veneta	0.485	-0.349	-0.086
	S. fruticosa	-1.457	-1.625	0.482
	Ctrl	-1.104	0.571	1.019
	SS	2.506	-1.236	-0.158
	WS	-1.402	0.665	-0.860

Table S4: Two-way analysis of variance (ANOVA) of stress treatments (ST), harvesting time (HT), and their interactions (STxHT) for the three halophyte species, for the 22 measured traits (fresh weight (FW), shoot water content (SWC), plant height (PH), number of branches (No.B), chlorophyll a (Chl.a), chlorophyll b (Chl.b), carotenoids (Caro), root sodium concentration (Na(r)), shoot sodium concentration (Na(s)), root chloride concentration (Cl(r)), shoot chloride concentration (Cl(s)), root potassium concentration (K(r)), shoot potassium concentration (K(s)), root calcium concentration (Ca(r)), shoot calcium concentration (Ca(s)), glycine betaine (GB), proline (PRO), total soluble sugars (TSS), malondialdehyde (MDA), hydrogen peroxide (H_2O_2), total phenolic compounds (TPC), total flavonoids (TF)). Significance codes: ns, (+), *, **, and *** mean, respectively, not significant and significant at $p \leq 0.1$, $p \leq 0.05$, $p \leq 0.01$ and $p \leq 0.001$

	S. europaea			*S. veneta*			*S. fruticosa*		
	ST	HT	STxHT	ST	HT	STxHT	ST	HT	STxHT
**FW**	<0.001***	0.070(+)	0.095(+)	<0.001***	0.003**	0.002**	0.002**	0.009**	0.897 ns
**SWC**	<0.001***	<0.001***	<0.001***	<0.001***	<0.001***	<0.001***	0.005**	0.002**	0.043*
**PH**	<0.001***	<0.001***	<0.001***	0.325 ns	<0.001***	0.182 ns	0.039*	0.420 ns	0.567 ns
**No.B**	0.008**	<0.001***	<0.001***	0.13 ns	0.002**	0.206 ns	0.224 ns	0.097(+)	0.444 ns
**Chl.a**	<0.001***	0.454 ns	0.133 ns	0.011*	0.921 ns	0.376 ns	0.203 ns	0.605 ns	0.782 ns
**Chl.b**	0.001**	0.452 ns	0.006**	0.012*	0.232 ns	0.009**	0.873 ns	0.397 ns	0.881 ns
**Caro**	0.009**	0.005**	0.038*	0.046*	0.668 ns	0.176 ns	0.495 ns	0.294 ns	0.977 ns
**Na(r)**	<0.001***	<0.001***	<0.001***	<0.001***	<0.001***	0.790 ns	<0.001***	<0.001***	<0.001***
**Na(s)**	<0.001***	0.719 ns	0.143 ns	<0.001***	0.324 ns	0.101 ns	<0.001***	0.265 ns	0.041*
**Cl(r)**	<0.001***	0.005**	0.005**	<0.001***	<0.001***	<0.001***	<0.001***	<0.001***	<0.001***
**Cl(s)**	<0.001***	0.378 ns	0.056(+)	<0.001***	0.195 ns	0.590 ns	<0.001***	0.579 ns	0.543 ns
**K(r)**	0.030**	<0.001***	0.056(+)	<0.001***	<0.001***	0.304 ns	<0.001***	0.879 ns	<0.001***
**K(s)**	<0.001***	<0.001***	0.164 ns	<0.001***	0.908 ns	0.010*	<0.001***	0.916 ns	0.246 ns
**Ca(r)**	<0.001***	<0.001***	<0.001***	<0.001***	<0.001***	<0.001***	<0.001***	<0.001***	<0.001***
**Ca(s)**	0.281 ns	0.092(+)	0.171 ns	<0.001***	0.314 ns	<0.001***	<0.001***	0.402 ns	0.620 ns
**GB**	0.349 ns	0.015*	0.501 ns	0.043*	0.459 ns	0.065(+)	0.563 ns	0.827 ns	0.003**
**PRO**	<0.001***	<0.001***	<0.001***	0.002**	0.025*	0.014*	0.015*	0.240 ns	0.993 ns
**TSS**	0.080(+)	0.696 ns	0.050(+)	0.026*	0.278 ns	0.632 ns	0.508 ns	0.379 ns	0.009**
**MDA**	<0.001***	<0.001***	0.627 ns	0.002**	0.539 ns	0.039*	0.015*	<0.001***	0.005**
**H_2O_2**	0.049*	0.007**	0.934 ns	0.083(+)	0.420 ns	0.072(+)	0.274 ns	0.021*	0.773 ns

**TPC**	0.479 ^{ns}	0.672 ^{ns}	0.737 ^{ns}	0.929 ^{ns}	0.446 ^{ns}	0.417 ^{ns}	0.181 ^{ns}	0.983 ^{ns}	0.319 ^{ns}
**TF**	0.176 ^{ns}	0.380 ^{ns}	0.993 ^{ns}	0.084 $^{(+)}$	0.209 ^{ns}	0.777 ^{ns}	0.026*	<0.001***	0.516 ^{ns}
